# The Prevalence of Pulp stones in Adult Patients of Shiraz Dental School, a Radiographic Assessment

**Published:** 2015-12

**Authors:** Shohreh Ravanshad, Shideh Khayat, Najmeh Freidonpour

**Affiliations:** a Dept. of Endodontic, School of Dentistry, Shiraz University of Medical Sciences, Shiraz, Iran.; b Dentist, Private practice, Rafsanjan, Iran.; c Dept. of Prosthodontics, School of Dentistry, Busheher University of Medical Sciences, Busheher, Iran.

**Keywords:** Pulp stones, Prevalence, Radiographic assessment

## Abstract

**Statement of the Problem:**

Pulp stones are calcifications found in the pulp chamber or pulp canals of the teeth. Its different prevalence in different population is a matter of concern.

**Purpose:**

This study aimed to assess the prevalence of pulp stones in a sample of Iranian population and to report its occurrence regarding gender, dental arch, tooth type and dental status.

**Materials and Methods:**

Dental records of patients who attended Shiraz Dental School were selected randomly. Only bitewing and periapical radiographs of maxillary and mandibular permanent posterior teeth were studied. Teeth were classified in the case of presence or absence of pulp stones, and the prevalence was analyzed in different gender, tooth types, dental arch, and dental status (intact, carious, or restored) groups. Statistical analysis was performed using *X^2 ^*test.

**Results:**

Of the 652examined subjects, 306 (46.9%) had one or more teeth with pulp stones. Of the 8244 posterior teeth examined, 928 (11.25%) had pulp stones in the pulp chamber. These pulp stones were detected in 76(37.6%) of males and 230 (51%) of females. The frequency of pulp stones among different teeth between maxillary and mandibular arches had almost a similar pattern. Among teeth demonstrating the condition, first molars were the most prevalent, followed by second molars. In maxillary molars the frequency of occurrence (26%) was higher than mandibular molars (18.7%). No Significant difference was found between dental status and pulp stones occurrence.

**Conclusion:**

The occurrence of pulp stones noted in this study was significantly higher in female, molar teeth than premolar and 1st maxillary molar than mandibular. There was no significant association between pulp stone and condition of the crown.

## Introduction

Pulp stones are calcified masses in the dental pulps of healthy, diseased, and even unerupted teeth.[1] They are located more often in the coronal than in the radicular portion of the pulp organ and can be seen free, attached, or embedded[2] Pulp stones were histologically classified by Kronfed into true or false forms; true stones containing irregular dentine and the false ones being degenerative pulp calcifications.[3]

Although etiological factors for pulp stone formation are not well understood; pulp degeneration, inductive interaction between epithelium and pulp tissue,[4] age,[5] circulatory disturbances in pulp,[6] orthodontic tooth movement,[7] idiopathic factors[8] and genetic predisposition[9] have been reported as causes of stone formation. The number of pulp stones in a single tooth has been detected to vary from one to 12 or more, while their size varies from small microscopic particles to large masses that might almost obliterate the pulp chamber.[10]

According to different studies, based on radiographic examination, the incidence of pulp stones in human dental pulps varies depending on type and study design. Tamse *et al.* reported that 20.7% of the examined teeth contained pulp stones; however, their study sample included lower premolars and first molars only.[11] Baghdady *et al.* studied the young adolescent population of Iraq and found that 19.2% of teeth that were radiographically examined had pulp stones[12] Hamasha and Darwazeh identified pulp stones in 22% of the first and second lower molars, in Jordanian adults,[13] and Ranjitkars *et al.* found pulp stones in 19.7% of the molars in a sample of Australian population.[14] In a radiographic assessment, the prevalence of denticles in Taiwanese was found to be 2.1%.[15] In 2009, the radiographs of two different groups of Turkish dental patients were examined for the prevalence of pulp chamber calcification and were reported to be about 5% in these Turkish individuals.[16-17]

Kazemizadeh Z *et al.* in 2011 calculated the prevalence of pulp stone in referred patients to Rafsanjan dental school and reported the prevalence of 20% in 800 patients.[18]

In Turkish Central Anatolian population, the prevalence of pulp stone was reported 27.8%,[19] while another radiographic survey of a Turkish population reported the prevalence of to be 15% in examined teeth.[20] Turkal M *et al.* detected pulp stone and reported a prevalence of 2.1%.[21]

Recently, the prevalence of pulp stone in Northern Indian Central Punjabi population and in Andhra Pradesh, India was reported 9.09% and 17.9% respectively,[22-23] and Javadzadeh A. *et al.* found pulp stone in 3.2% of the teeth in patients referred to Guilan Dental School.[24]

Although the exact etiology of pulp calcification is unknown, it is clearly shown that the frequency of occurrence of pulp stones has been reported to increase with age.[16-19,24] Gender in some studies did not demonstrate any difference in occurrence,[7,16,18,20,23-24] whereas other studies have found females to have more pulp stones than males.[11,15,19,21-22,27]

This study was conducted concerning the very limited published data regarding the prevalence of radiographically visible pulp calcifications in the pulp chambers of permanent teeth in Iranian population. In this study, the prevalence of pulp calcification of permanent posterior teeth was evaluated with in a group of male and female subjects using radiographic examinations. Its relationship with the tooth crown condition (intact, carious, and restored) was also investigated. 

## Materials and Method

In this retrospective study, from 5500 dental records available in periodontal department archives, a random sample of 926 records were selected. All of these dental patients had been treated at School of Dentistry, Shiraz University of Medical Sciences, Iran, during the period of 2003 to 2009. The medical examination of the patients was not contributory. Patients less than 18 years of age at the time of radiographic examination were excluded (195 subjects); patients records with radiographs of poor quality (inadequate exposure or processing faults causing scoring difficulties) were also excluded from the study (79 subjects).The final sample consisted of 652 records, all of which had radiographs that were adequate to allow determination of the presence or absence of pulp stones. Of the 652 patients 202(30.9%) were male and 450(69%) were female. The mean age was 34.4 years (standard deviation ± 9.575), ranging from 18 to 70 years. Of the 652 patients, a total of 2608 radiographs were scrutinized and a total of 8244 teeth were imaged in the radiographs. Only bitewing and periapical radiographs of maxillary and mandibular permanent premolar and molar teeth (excluding third molar) were studied. All radiographs were inspected by two experienced examiner using a magnifying lens and an X-ray viewer in darkened room, using a light box with an even diffuse light source, and with peripheral light blocked out. A tooth was categorized as having a pulp stone only when a clear radiopaque mass could be seen in the pulp chamber and scored as present or absent. The dental status of each tooth was categorized as unrestored and intact, or restored and/or carious. The size, shape, and number of pulp stones were not evaluated and scored. The information obtained from the radiographic examination was recorded on a special sheet prepared for this study. To check the reliability of the radiographic examination, the examiner re-evaluated the radiographs from a sample of 100 dental records two months after the first examination; both examinations were consistent.

Statistical analysis of the data was performed using SPSS software. Frequency distribution of teeth with pulp stones was calculated. Chi-square analysis was used to compare the frequencies of occurrence of pulp stones between males and females, tooth type, jaws, and dental status. Statistical significance for the analysis of the results was set at the 5percent probabilities (*p*< 0.05).

## Results

Of 652 subjects, 306 (46.9%) had one or more posterior teeth that contained pulp stones which varied from one to ten teeth in each subject, while it was not found in 346(53.1%) subjects (Table 1). These were detected in 76 (37.6%) of 202 males and 230 (51%) of 450 females. The overall difference in distribution between genders was statistically significant (*p*= 0.001).

**Table 1:** Frequency of number of teeth with pulp stones in subjects

**Number**	**0**	**1**	**2**	**3**	**4**	**5**	**6**	**7**	**8**	**9**	**10**
Frequency	346	73	82	43	47	25	16	10	8	1	1
Percent	53.1	11.2	12.6	6.6	7.2	3.8	2.5	1.5	1.2	0.2	0.2

Pulp stones were observed in 928 of 8244 examined teeth, with a prevalence of 11.25%. Pulp stones were found in only 20 (0.47%) of the 4226 premolars and in 908 (22.6%) of 4018 molars, with the differences in occurrences being statistically significant (*p*< 0.05). Frequency of pulp stones in premolar and molar teeth on the basis of location are summarized in Table 2.

**Table 2:** Frequency of pulp stones of maxillary and mandibular premolar and molar teeth in adult patients

**Tooth type**	**Maxilla**		**Mandible**		**Sum**	
	**Total**	**With pulp stone**	**Total**	**With pulp stone**	**Total**	**With pulp stone**
	**N**	**N (%)**	**N**	**N (%)**	**N**	**N (%)**
P1	1018	3(3)	936	6(0.6)	1954	9(0.46)
P2	1162	6(0.5)	1110	5(0.45)	2272	11(0.48)
SUM P	2180	9(0.4)	2046	11(0.5)	4226	20(0.47)
M1	1035	372(36)	857	238(27.7)	1892	610(32.2)
M2	1070	178(16.6)	1056	120(11.3)	2126	298(14)
SUM M	2105	550(26)	1913	358(18.7)	4018	908(22.3)
Total	4285	559(13)	3959	369(9.32)	8244	928(11.25)

P1: First premolar             P2: Second premolar            M1: First molar            M2: Second molar

The frequencies of pulp stones were higher in first molars (32.2%) than in second molars (14%). Total occurrence of pulp stones was higher in maxillary molars (26%) than in mandibular molars (18.7%) but significant for first molar teeth only (36% vs. 27.7%) (Table 3) The molar teeth with pulp stones were examined for intact and restoration and /or caries. 2629 (65.4%) of the 4018 molars were unrestored and intact (sound) and 1389 (34.6%) were restored and/or carious which reflect that there were factors that could cause pulp irritation. 560 (21.3%) of sound teeth and 348 (25%) of restored and/or carious teeth had pulp stone. There was no significant association between dental status and pulp stone occurrence.

**Table 3:** Prevalence of pulp stones in molar teeth with different crown status

**Tooth type**	**Unrestored and Intact**			**Restored and/or Carious**	
		**Sum**	**Teeth with pulp stones**	**Sum**	**Teeth with pulp stones**
		**N**	**N (%)**	**N**	**N (%)**
Maxillary					
M1	1035	567	261 (46)	468	111 (23.7)
M2	1070	890	127 (14.2)	180	51 (28.3)
Mandibular					
M1	857	413	109 (26.4)	443	129 (29)
M2	1056	759	63 (8.3)	297	57 (19.2)
Total	4018	2629	560 (21.3)	1389	348 (25)

M1: First molar            M2: Second molar

## Discussion

In the current study, pulp stones observation was based on the bite-wing and periapical radiographs, since dental radiographs are the only means for non-invasive evaluation. In bite-wing radiographs, one can obtain more standard picture because it is perpendicular to the long axis of the tooth, while contrast distortion could occur in the periapical radiographs, yet no statistical differences were observed between these two techniques.[4] One must note that all of the pulp's calcifications would not be demonstrated by radiographs. A calcified body less than 200µm cannot be detected in radiographs; therefore, a very small sized pulp stone cannot be viewed on radiographs.[4, 16]

The prevalence of pulp stones recorded in the examined teeth of this study was 11.25% which is almost similar with that reported recently by Ranjitkar in Australian population,[14] yet it was lower than the values reported by Hamasha and Darwazeh,[13] Baghdady *et al.*[12] and Tamse *et al.*[11]

In 1982, Tamse *et al.*,[11] in 2009 Senser *et al.*,[16] in 2012 Colak *et al.*,[19] and in 2013 Turkal *et al.*[21] reported that pulp stones were more common in females than males. The prevalence of pulp stones found in the current study is similar and statistically confirms those findings. In Bagdady’s study, the incidence of pulp stones in male group was slightly higher than in female group, but the difference was not statistically significant and it was in agreement with the finding of other studies that reported no significant difference between genders.[6,13-14,17-18,20,23] The finding that the occurrence of pulp stones tended to be higher in molars (20.3%) than in premolars (0.47%) is consistent with earlier reports.[11-14,16-17,19] In this study, the prevalence of pulp stones was found to be higher in the first molars (32.2%) than in the second molars and premolars, which confirms the result of previous investigations.[11-14,18, 20, 22] The first molar is the first posterior permanent tooth that erupts and contains more pulp stones than others. In our study, higher frequencies of pulp stones found in maxillary molars are in accordance with Ranjikar’s, Senser’s, Colak’s, Turkal’s, Bains’s, and Javadzadeh’s studies. However, in other studies, similar frequencies in both arches,[4, 26] or higher occurrence in mandibular teeth have been reported.[12-13,26]

The higher incidence of pulp stones in the molars might be due to earlier eruption of the molars compared to premolars, exposing molars for more possible degenerative changes. Furthermore, first molar is the largest tooth in the arches with a large pulp chamber, a greater amount of pulp tissue, and a better blood supply,[1,11] those factors which may contribute to the conditions that precipitate calcifications.

Our study did not reveal a significant association between presence of pulp stones and the condition of tooth crown (sound, carious and/or restored). This was the same as Tamse *et al.*,[11] Baghdady *et al.*[12] and Gulsahi *et al.*[17] reports, and also in agreement with Ranjikar’s[14] and Senser’s[16] studies that reported carious and restored teeth displayed higher prevalence of pulp stone than intact molar teeth.

Pulpal pathology is unlikely to be the only etiological factor for formation of pulp stone since even in very young teeth and developing tooth germs, the presence of pulp stones was reported.[4]

Pulp stones generally have no clinical significance. However, they might obstruct the pulp chamber and root canals, causing difficulties during root canal treatment.[28] Such clinical difficulties may be eliminated by magnification; good access and using appropriate instruments during endodontic therapy.

## Conclusion

The features of pulp stones noted in current survey may provide additional information regarding the dental morphological features of Iranian adults. This study showed that female patients were more predisposed to developing pulp stones than male patients. The pulp stones were found to occur more in maxillary than in mandibular teeth, with a higher prevalence in first molars compared with other posterior teeth. Our study does not show a positive relationship between pulp stone incidence and intact, carious and/or restored teeth. 
